# Clinical efficacy of a new type of Sports Rehabilitation Therapy Bed

**DOI:** 10.12669/pjms.40.6.7871

**Published:** 2024-07

**Authors:** Duoduo Yu, Xiaobing Luo, Piming Gao

**Affiliations:** 1 Duoduo Yu, Department of Orthopedics, Sichuan Province Orthopedic Hospital, Chengdu 610041, Sichuan, China; 2 Xiaobing Luo, Department of Orthopedics, Sichuan Province Orthopedic Hospital, Chengdu 610041, Sichuan, China; 3 Piming Gao, Department of Orthopedics, Sichuan Province Orthopedic Hospital, Chengdu 610041, Sichuan, China

**Keywords:** Sports rehabilitation therapy bed, Precise stretching, Bare-handed stretching, Clinical study

## Abstract

**Objective:**

To prove that the “sports rehabilitation bed” is a device aimed at improving the precision of stretching, which can help to reduce the difficulty of rehabilitation therapy, cut down the manpower input of rehabilitation therapy, and shorten the therapy duration as well.

**Methods:**

This was a clinical comparative study. Twenty patients who underwent stretching therapy in Sichuan Province Orthopedic Hospital from June 2020 to August 2020 were randomly selected to carry out a control study on both lower extremities. The experimental group was given sports rehabilitation bed to assist rehabilitation therapy, while the control group was given conventional bare-handed stretching rehabilitation therapy. The stretching angle, stretching value, and the effective rate of stretching therapy between the two groups to analyze the clinical value of the new sports rehabilitation therapy bed.

**Results:**

The stretching angle in the experimental group when using the sports rehabilitation therapy bed for stretching was lower than the conventional bare-handed stretching in the control group (T<0, P=0.05), with a statistically significant difference; the stretching values of the experimental group were lower than those of the control group(P<0.01), with a statistically significant difference. Moreover, the response rate of stretching therapy in the experimental group was lower than that in the control group(P<0.05), with a statistically significant difference.

**Conclusion:**

Sports rehabilitation therapy beds can results in the advantages of effectively preventing iatrogenic injury in the process of stretching, and providing a more accurate and convenient stretching therapy method than the current commonly used bare-handed stretching for sports rehabilitation and intervention.

## INTRODUCTION

Muscle stretching, whether it is active or passive stretching, is one of the conventional rehabilitation therapies for musculoskeletal diseases. The following rehabilitation methods are commonly used:

### Proprioceptive neuromuscular facilitation (PNF)

a method that muscles are forced to contract to induce reflex self-inhibition, and then the muscles are relaxed by stretching.

### Static stretching

the method of stretching muscles to the extreme point and standing still.

### Dynamic stretching

the method of slowly moving the joint to the stretching position.

### Elastic-shock stretching

the method of making limbs move from the initial posture to the stretching posture by rebound movement.

In the process of diagnosis and treatment, the above methods are used alone or in combination. In any case, their rationale is to stretch the shortened or contractured tissues and lengthen them, so that subjects can regain the extensibility of soft tissues around joints, reduce muscle tension, improve muscle excitability and improve or restore the normal range of motion of joints. For example, the incidence of patellofemoral pain syndrome (PFPS) in the general population is as high as 22.7%,^1^ which is significantly related to the lack of flexibility of quadriceps femoris (QF).^2^ In this regard, stretching training of quadriceps femoris can effectively improve the flexibility of quadriceps femoris. However, the quadriceps femoris is a cross-joint muscle. In the process of knee extension, the antagonistic muscle of quadriceps femoris (AM) are biceps femoris (BF), semimembranous muscle (SM) and semitendinosus. During hip flexion, the antagonistic muscles are biceps femoris, semitendinosus, gluteus maximus (GM) and piriformis muscle (PM). The hip can only be effectively stretched to this muscle group if it is fully extended posteriorly and then flexed. In view of the linkage relationship between pelvis and lumbar vertebrae, when hip is extended, there will be corresponding pelvic forward extension and lumbar vertebrae backward extension if pelvic fixation is not sufficient, which will increase the compression force at the back of the waist, thus causing lumbar injuries. Therefore, the antagonistic action of antagonistic muscles should be limited during stretching. Besides, muscle stretching is also one of the commonly used methods in the field of sports training, which is widely used in improving sports performance.

In most studies, dynamic stretching is mainly used to warm up before sports, which can activate muscles and enhance their maximum strength and explosive power,^3^ while static stretching is mainly used to recover after sports, which can help athletes improve the stiffness, elasticity and viscosity of myofascial,^4^ relieve pain,^5^,^6^ accelerate fatigue elimination and reduce the incidence of muscle injury.^7^ Still, a few scholars dispute this. Some foreign scholars have pointed out that many studies on stretching function lack the description of stretching details,^8^ and only describe the stretching method, time and strength, such as “static stretching method, stretching for 30 seconds each time, repeating three groups, and resting for 30 seconds between groups”,^9^ in which the strength is mostly evaluated by subjective feelings, such as “stretching with mild discomfort”.^10^ Whether or not there is a unified standard of body position during stretching, the difference in operator’s proficiency and the quantitative evaluation of stretching degree may all affect the research results. But at present, the articles and textbooks on stretching do not involve this aspect.

There are still some problems in the research and clinical application of stretching, mainly manifested in the standardization of posture, the lack of accuracy of movements, and the lack of quantitative evaluation of stretching strength and angle, which may affect the rigor of research and clinical effect. Therefore, it is necessary to design a sports rehabilitation therapy bed to solve the above problems. The sports rehabilitation therapy bed cannot only strictly regulate the posture of the subjects during stretching, but also ensure the accuracy and effectiveness of stretching, and reduce the workload of the therapist. For inexperienced therapists, the sports rehabilitation therapy bed can help them make up for their own shortcomings and achieve a better stretching effect by using the treatment bed.

## METHODS

This was a clinical comparative study. Twenty patients who underwent stretching therapy in Sichuan Province Orthopedic Hospital from June 2020 to August 2020 were divided into two groups by the control method of left and right lower limbs. All patients were divided into two groups according to the random number method: the experimental group (20 subjects were treated with left lower limb) and the control group (20 subjects were treated with right lower limb). The subjects in the experimental group were given muscle stretching therapy on the bed of sports rehabilitation therapy, while those in the control group were given bare-handed muscle stretching therapy. Meanwhile, before receiving therapy, the subjects underwent a muscle length test of rectus femoris, and measured the height h(cm) from the marked point of lateral femoral condyle to the examination bed, the angle between femur and bed surface, and the knee flexion angle using the Thomas test method. Then, the sports rehabilitation therapy bed was placed in the stretching posture of rectus femoris, and the rectus femoris stretching intervention was performed on the subjects. After that, the above-mentioned height and two angles were measured again, and the differences before and after intervention were compared.

### Ethical Approval

The study was approved by the Institutional Ethics Committee of Sichuan Province Orthopedic Hospital (No.:KY2022-029-001; Date: October 19, 2022), and written informed consent was obtained from all participants.

### Inclusion criteria:

- Subjects aged 20-30 years
- Subjects with complete medical history information, no history of neuromuscular or skeletal system injury, and high compliance;
- Subjects without motor system disease within six months prior to participation in the intervention treatment;
- Subjects who did not receive any intervention and treatment for a diagnosis of skeletal muscle, bone and joint within six months prior to participation in the intervention treatment;
- Subjects who agreed to the study protocol and signed the informed consent form.

### Exclusion criteria:

- Subjects who did not accept the study protocol;
- Subjects who did not meet the inclusion criteria ;
- Subjects who did not follow the prescribed protocol;
- Subjects who failed to complete the trial for various reasons (e.g., adverse events, patient missed visits);
- Subjects who voluntarily withdrew their informed consent;
- Subjects who developed serious adverse reactions and discontinued the trial by the combined decision of the subjects or the investigator.

**Fig.1:**
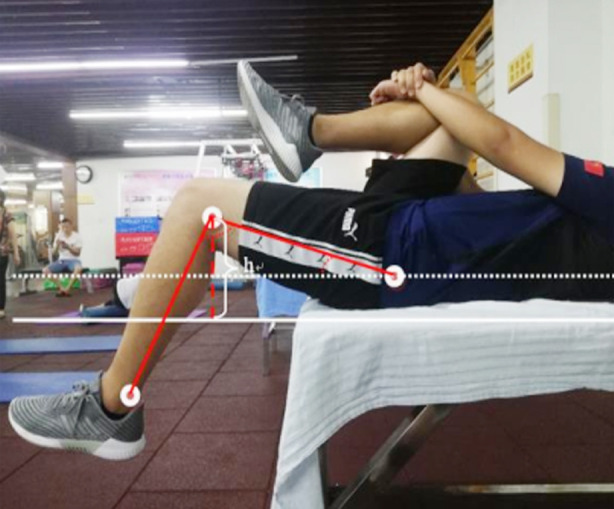
Thomas test.

sEMG (electrode disc diameter of 1cm, distance between two electrode centers of 2cm) was recorded by bipolar recording method. Electrode sheets were affixed according to the operation manual of the MegaWin ME6000 sensor. Every three electrode sheets were affixed to the most eminence of muscle abdomen of quadriceps femoris (target muscle), biceps femoris (antagonistic muscle) and tibialis anterior (synergistic muscle)) respectively. Besides, the connecting direction of the electrode sheets was parallel to the direction of muscle fibers. The EMG signals of quadriceps femoris, biceps femoris and tibialis anterior muscle were collected by a 16-channel Mega ME6000 surface electromyography system when the subjects stretched on the sports rehabilitation therapy bed for the first time and stretched by hand respectively. The degree of the knee joint of the subjects was extracted from the beginning of stretching to the onset of the stretching sensation. Furthermore, the EMG signals 30 seconds after the onset of the stretching sensation were recorded, with the Average electromyography (AEMG) as the index, and the obtained values were standardized. The two groups of patients were followed up for six months, and the follow-up work of all patients was completed by the same group of surgeons.

### Statistical analysis:

All data in this study were statistically analyzed by SPSS21.0 software, and measurement data were expressed as(*χ̅*±*S*). Paired t test was used for intra-group comparison, and an independent sample t test for inter-group comparison. The statistical data were expressed as rate (%), and χ^2^ test was used for the comparison of rates, with P<0.05 indicating a statistically significant difference.

## RESULTS

The comparison of general data between the experimental group and the control group showed no statistical significance(P>0.05). Thigh circumferences on both sides of each subject were comparable, with no statistically significant difference(P>0.05), as shown in Table-I.

**Table-I:** Comparative analysis of general data of the two groups (*χ̅*±*S*).

N	Gender (male/female)	Age	Body weight (kg)	Height (cm)	Thigh circumference (left/right)
20	9/11	26.8±7.56	62.3±5.6	160.2±9.55	Left: 43.65±0.815
					Right: 43.55±0.771

**Table-II:** Comparative analysis of the stretching angle.

Group	Stretching angle
Experimental group (sports rehabilitation therapy bed stretching)	103.04°±13.01°
Control group (conventional bare-handed stretching)	108.33°±12.27°
*T*	-2.046
*P*	0.050

**Table-III:** Comparative analysis of muscle stretching values between the two groups.

Muscle/Group	Experimental group (sports rehabilitation therapy bed stretching)	Control group (conventional bare-handed stretching)	P	T
Quadriceps femoris	2.90±1.373	5.70±4.426	0.019	-2.564
Biceps femoris	3.60±2.501	10.15±6.588	0.001	-4.048
Tibialis anterior	2.85±0.875	5.50±3.591	0.006	-3.118

**Table-IV:** Comparative analysis of an active range of motion before and after stretching between the two groups.

Group	Active range of motion before stretching	Active range of motion after stretching
Experimental group (sports rehabilitation therapy bed stretching)	120-142	122-146
Control group (conventional bare-handed stretching)	120-140	122-145
*T*		
*P*		

**Table-V:** Comparative analysis of the response rate of stretching therapy between the two groups.

Group	Marked response	Moderate response	Overall response rate*
Experimental group	15(75%)	5(25%)	20(100%)
Control group	11(55%)	9(45%)	20(100%)
*χ* ^ 2^	-	-	
P	-	-	

The stretching angle in the experimental group when using the sports rehabilitation therapy bed for stretching was lower than the conventional bare-handed stretching in the control group (T<0, P=0.05), with a statistically significant difference.

In the experimental group, quadriceps femoris, biceps femoris and tibialis anterior muscle were stretched by sports rehabilitation therapy bed, and their stretching values were lower than those of the control group(T<0). The stretching values of the experimental group were lower than those of the control group (P<0.01), with a statistically significant difference.

The active range of motion before and after stretching in the experimental group was lower than that in the control group, with a statistically significant difference(P<0.05). The response rate of stretching therapy in the experimental group was lower than that in the control group (P<0.05), with a statistically significant difference.

## DISCUSSION

These results suggest that, in sports intervention and rehabilitation therapy, due to the limitation of the subjects’ postures and the limitation of joint activities through the fixed movable foam axis, the sports rehabilitation therapy bed minimizes the participation of interference factors such as antagonistic muscles and synergistic muscles, which is more accurate than the traditional freehand stretching technology. There is also the advantage of stretching the target muscle at a relatively low angle.

Muscle stretching is a physical therapy, which is a kind of sports therapy to improve the effectiveness of rehabilitation therapy mainly by exercising the most vulnerable muscle groups. Stretching therapy can effectively promote local blood circulation of muscles, accelerate the dissipation and absorption of inflammatory substances and metabolites, and repair damaged soft tissues. Still, it cannot only improve muscle tension, elastic energy and extensibility, but also prevent muscle contracture, increase muscle length and relieve pain, thus improve the therapeutic effect of rehabilitation therapy and promote recovery.^11^-^15^

In the current clinical rehabilitation muscle stretching therapy, it mainly relies on the strength of the therapist to help the subjects fix or move their limbs. What’s more, both the stretching angle and the stretching sensation are described by the subjective proprioception of the subjects, which cannot guarantee the standardization of the posture and the accuracy of movements, but also increase the workload of the therapist. However, if the stretching therapy relies entirely on the therapist’s experience, the risk of injury or re-injury to tissues like bones and joints will be increased due to the existence of individual differences. The results of this study show that for the subjects in the experimental group treated with a new type of self-developed sports rehabilitation bed, their efficacy after treatment was significantly higher than that of the control group with conventional freehand stretching.

### Limitations

It includes the modest sample size and the lack of long-term outcomes observation. In view of this, further improvements will be made in future research to make more scientific research results.

## CONCLUSIONS

Thus, the therapeutic effect of precise stretching is achieved with this treatment modality.. All this indicate that the new sports rehabilitation therapy bed is superior to the bare-handed stretching therapy, which is of great clinical promotion significance.^16^-^20^

### Authors’ Contributions:

**DY:** Carried out the studies, participated in collecting data, drafted the manuscript, are responsible and accountable for the accuracy and integrity of the work.

**XL:** Performed the statistical analysis and participated in its design.

**PG:** Participated in acquisition, analysis, or interpretation of data and draft the manuscript.

All authors read and approved the final manuscript.
